# Early‐Life Exposure to Pulsed LTE Radiofrequency Fields Causes Persistent Changes in Activity and Behavior in C57BL/6 J Mice

**DOI:** 10.1002/bem.22217

**Published:** 2019-09-15

**Authors:** Kerry A. Broom, Richard Findlay, Darren S. Addison, Cristian Goiceanu, Zenon Sienkiewicz

**Affiliations:** ^1^ Centre for Radiation, Chemical and Environmental Hazards Public Health England Chilton Oxfordshire UK; ^2^ Physics Group, EMFcomp Limited Harwell Campus Harwell Oxfordshire UK; ^3^ Department of Environmental Health, National Institute of Public Health Regional Center Iasi Iasi Romania

**Keywords:** electromagnetic fields, locomotion, activity, brain, rodent

## Abstract

Despite much research, gaps remain in knowledge about the potential health effects of exposure to radiofrequency (RF) fields. This study investigated the effects of early‐life exposure to pulsed long term evolution (LTE) 1,846 MHz downlink signals on innate mouse behavior. Animals were exposed for 30 min/day, 5 days/week at a whole‐body average specific energy absorption rate (SAR) of 0.5 or 1 W/kg from late pregnancy (gestation day 13.5) to weaning (postnatal day 21). A behavioral tracking system measured locomotor, drinking, and feeding behavior in the home cage from 12 to 28 weeks of age. The exposure caused significant effects on both appetitive behaviors and activity of offspring that depended on the SAR. Compared with sham‐exposed controls, exposure at 0.5 W/kg significantly decreased drinking frequency (*P* ≤ 0.000) and significantly decreased distance moved (*P* ≤ 0.001). In contrast, exposure at 1 W/kg significantly increased drinking frequency (*P* ≤ 0.001) and significantly increased moving duration (*P* ≤ 0.005). In the absence of other plausible explanations, it is concluded that repeated exposure to low‐level RF fields in early life may have a persistent and long‐term effect on adult behavior. Bioelectromagnetics. 2019;40:498–511. © 2019 The Authors. Bioelectromagnetics Published by Wiley Periodicals, Inc.

## INTRODUCTION

People are increasingly exposed to a broad spectrum of radiofrequency (RF) fields from an array of sources operating from a few hundred MHz to a few GHz. Sources include mobile and cordless phones, Bluetooth devices, and Wi‐Fi [Sienkiewicz et al., 2017]. Despite much research, there are still gaps in knowledge about the potential of low‐level RF fields to cause biological effects, and there are concerns that these exposures may have long‐term effects on human health [SCENIHR, 2015]. In addition, the ubiquitous nature of our exposure to these RF fields means that, even if the risk to individuals is low, a substantial number of people among the population could experience health effects [Kheifets et al., 2001; WHO, 2010].

Since the development of mobile cellular telephony, adults and children have been exposed to prolonged low‐level RF fields from base stations and to acute, localized exposures when the phone handsets are used. Children have been assumed to be more sensitive than adults to these exposures, due to the greater absorption of RF fields in the tissues of the head, and a longer lifetime of exposure IEGMP [2000]. The main potential risks for children are likely to be associated with delays in the development and maturation of the central nervous system, and adverse effects on immune system and other critical organs [IEGMP, 2000; Kheifets et al., 2005; Leung et al., 2011].

Few human data are available, but Divan et al. [2008, 2012, 2011] have reported that behavior in young children may be affected by their mothers’ mobile phone use during pregnancy, and emotional, communication, and motor skills have also been reported to have been affected [Sudan et al., 2016; Papadopoulou et al., 2017]. Other studies have shown that there is no association with mobile phone use and behavioral problems [Vrijheid et al., 2010; Guxens et al., 2013]. A few studies reported behavioral problems associated with mobile phone use. Maternal mobile phone use during pregnancy was assessed, as well as use by the child. Disruptive behavior including temper tantrums, disobedience, and attention deficit hyperactivity disorder were studied and an elevated risk of behavioral problems was associated with both maternal and child phone use [Divan et al., 2008, 2012]. In addition, a study has reported that maternal cell phone use during pregnancy may be associated with an increased risk for hyperactivity in children, although caution in interpretation is needed due to confounding factors [Birks et al., 2017]. Further studies investigating mobile phone use during pregnancy and by young children are currently underway as part of the EU‐funded Geronimo project [GERoNiMO, 2018]. The avoidance of possible cognitive deficits has helped to underpin the present precautionary advice regarding the use of mobile phones by children in many countries, including the UK [Department of Health, 2011]. Currently, there is no convincing evidence that RF exposure below the guidelines recommended by the International Commission on Non‐Ionizing Radiation Protection (ICNIRP) can cause health effects [Sienkiewicz et al., 2016]; however, the technology is still relatively new and concerns remain.

While it has long been recognized that exposure of pregnant animals to RF fields at thermal levels (generally above a specific absorption rate of 4 W/kg that causes a sustained increase in maternal core body temperature of 1°C or more) [ICNIRP, 1998] may cause teratogenic effects and disrupt the development of offspring, the possibility that more subtle morphological, behavioral, or cognitive changes may occur following extended low‐level exposures (that do not cause elevation in body temperature) during early life cannot be ruled out [Aldad et al., 2012; Haghani et al., 2013; Zhang et al., 2015]. Although a study by Poulletier de Gannes et al. [2012] using 2,450 MHz Wi‐Fi signals at SARs of 0.08, 0.4, and 4 W/kg did not report any RF field‐dependent changes on functional development (including body mass and food consumption), Kumlin et al. [2007] reported improvements in learning in a water maze task after exposure of rats to 900 MHz GSM signals at 0.3 or 3 W/kg.

Few studies have investigated the biological consequences of exposure to fourth generation of mobile phone technology (called 4 G or long term evolution, LTE) signals associated with smartphones. Those studies have focused on effects on the brain during and after acute exposure in humans [Yang et al., 2017; Vecsei et al., 2018] or effects on spermatogenesis after longer exposure in rodents [Oh et al., 2018]. This research aims to study the possibility that early‐life and prenatal exposure in mice may cause effects on innate behaviors that persist in adult animals. It is unclear whether any effect on these innate behaviors, such as food and water consumption and locomotor activity, would be detrimental or beneficial.

To this end, mice were exposed to pulsed 1,800 MHz fields for 30 min/day, 5 days/week from late in gestation until weaning. The whole‐body average specific energy absorption rate (SAR) used was 0.5 or 1 Wkg. These time points are intended to mimic young life exposures during a period of crucial brain and hippocampal development. As little is known about the effect of exposure on behavioral, physiological, and consummatory behavior, assessments of drinking and feeding behavior as well as locomotor and rest‐activity in the exposed mice were compared with identically treated sham‐exposed mice for 17 weeks.

## METHODS

### Animals

**Figure 1:**
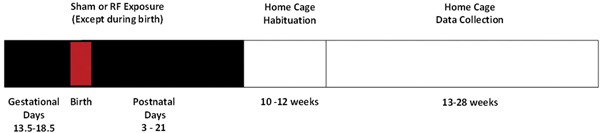
Experimental design. Animals were exposed to pulsed 1,846 MHz radiofrequency (RF) fields for 30 min/day, 5 days/week from gestation days 13.5–18.5 and from postnatal days 3 to day 21 (weaning) at a whole‐body averaged specific energy absorption rate (SAR) of 0.5 or 1 W/kg using a Gigahertz transmission electromagnetic cell (GTEM). Sham exposures consisted of the same procedures except that the electric field strength in the GTEM was reduced to 0. Two weeks were allowed for habitation to the home cages before data collection from 13 to 28 weeks of age.

C57BL/6 J mice were used for these investigations as they are exceptional breeders and are widely used in behavioral studies. Naïve males and females at 6–8 weeks of age were obtained from a commercial supplier (Envigo RMS, Bicester, UK). One week after arrival, three females were mated overnight with one male (taken as gestational day 0.5), and pregnant animals were randomly assigned to a sham (control) or treatment group. Treatment groups were as follows: 0.5 or 1 W/kg. Except during exposures and behavioral testing, the pregnant mice (or dam and litter) were housed in individual polycarbonate cages in a ventilated cabinet (SCANBUR, Copenhagen, Denmark) under controlled environmental conditions (19–21°C; relative humidity 45–65%) with a 12‐h light/dark cycle from 06:00 h to 18:00 h. Standard laboratory diet and water were freely available, and bedding was provided by commercial aspen wood shavings and shredded tissue paper. All litters were culled to six offspring on the day of birth. To avoid the impact of variation in estrous cycle, only male mice were used in this experiment, with no more than one animal from each litter in any treatment group. The experiment was replicated twice. For measurement of body mass *n* = 3, but for behavioral endpoints all treatment groups consisted of *n* = 5 or 6 except for weeks 14, 21, and 28 where *n* = 3 due to data loss as summarized in Table 1 (brought about by power failure or equipment malfunction resulting in the inability to capture video‐tracking data) The experimental design is shown in Figure 1.

**Table 1:** Summary of the Number of Mice Per Exposure Group at the Time‐Points of Behavioral Observation in The PhenoTyper Cages

	Number of mice per exposure group		
Timepoint of behavioural observation (animal age, weeks)	Sham	0.5 W/kg	1 W/kg
12	6	6	6
13	6	6	6
14	3	3	3
15	5	6	6
16	5	6	6
17	5	6	6
18	5	6	6
19	5	6	6
20	5	6	6
21	3	3	3
22	5	6	6
23	5	6	6
24	5	6	6
25	5	6	6
26	5	6	6
27	5	6	6
28	3	3	3

The animals’ health status was monitored throughout the experiments by a health surveillance program according to guidelines of the Federation of European Laboratory Animal Science Association (FELASA). The mice were free of all viral, bacterial, and parasitic pathogens listed in the FELASA recommendations, except for a few cases of *Tritrichomonas* species, *Helicobacter* species, and mouse norovirus within the facility. All experiments were performed in accordance with the UK Animals (Scientific Procedures) Act, 1986, and all protocols were approved by the Animal Welfare Ethical Review Body at the Center for Radiation, Chemical and Environmental Hazards, Public Health England.

### Exposures

A realistic downlink (DL) LTE signal was generated at a frequency of 1,846 MHz using an appropriate software package (Agilent Signal Studio N7624B, LTE 3GPP FDD Release 9; Keysight Technologies, Wokingham, UK). This signal was fed into a signal generator (Agilent E4438C; Keysight Technologies) and amplified using a broadband amplifier (175S1G4; Amplifier Research UK, Milton Keynes, UK), and then in a GTEM cell (Model 5311; EMCO, Austin, TX). The electric field strength was monitored using an electric field probe (HI‐6105; ETS‐Lindgren, Cedar Park, TX), which had an UKAS‐accredited calibration by the National Physical Laboratory (NPL) (Teddington, UK).

The animals were exposed to LTE‐modulated RF field levels that produce the desired SAR levels of 0.5 and 1 W/kg, except the fetuses where the SAR in a particular fetus varied in a rather large interval for a SAR of 0.5 or 1 W/kg in the mother (as explained below). SAR exposure levels were chosen to reflect realistic exposures and previous published research and did not cause a significant elevation in body core temperature in experimental animals. Therefore, the SAR target levels were chosen to be below the known level of 4 W/kg which induces an increase of body temperature of about 1°C. SAR levels of 0.5 and 1 W/kg should not cause hypothermia in mice which adversely affects the behavior of the animals. On the contrary, these levels are about the same level compared with the ones mentioned in the literature describing studies on non‐thermal effects (see references in Introduction). In conclusion, the two exposure levels were chosen to be below those leading to a heavy thermal load on mice, but not too low (only several times below 4 W/kg) to allow emphasizing possible effects in a reasonable time scale as the one in our study. As long as SAR levels and distribution are dependent on body size, geometry, and posture, the use of computational dosimetry allowed for the calculation of the required value of the external incident electric field that had to be set to produce a desired SAR value in the animals. In summary, a high‐resolution, anatomically realistic model of a pregnant C57BL mouse with six fetuses was created (section views are shown in Fig. 5) to study fetus field exposure while within the abdominal cavity of the adult mouse. Additionally, heterogeneous models of young mice in various stages of development were produced to investigate the effects of exposure in early life.

The finite‐difference time‐domain method (FDTD) was used to simulate the interaction of a 1,846 MHz field with the tissues in the mouse models and to calculate both the magnitude and spatial distribution of the SAR. SAR distribution within the pregnant model is also shown in Figure 5. Exposures to the left, right, and front of the mouse models were simulated to determine the electric field strengths required to produce SAR levels of 0.5 and 1 W/kg within the animals during both prenatal and early‐life exposure. The spatial resolution of these calculations varied between 0.1 and 0.5 mm resolution depending on the size of the animal under investigation.

The whole‐body averaged SAR calculated in the mother and fetus was dependent on field incidence and mother/fetus position. For prenatal exposure, if the electric field is kept at a value that produces a whole‐body averaged SAR of 0.5 or 1 W/kg in the mother, the SAR in a particular fetus varies between 0.125–1.39 and 0.249–2.79 W/kg, respectively. The calculated variation of SAR among fetuses is in line with the expected distribution of SAR in the body of a mother. For a given incidence of the field and a specific mother position, the SAR in a particular fetus is much dependent on its position inside its mother, and that leads to various levels of exposure in different fetuses that spread over a decade.

The pups were free to move during exposure (see below), but they tended to nest in a group and so “mass of pups” (i.e. six pups in a group) SAR calculations were performed. The “mass of pups” heterogeneous model was linearly scaled as a group to represent growing pups of various sizes, from a single pup mass of 0.9–29 g. Calculations of electric field strength required to produce the SAR levels of 0.5 and 1 W/kg were carried out for frontal exposure of the group and emphasized the dependence of the efficiency of RF energy absorption with the mass of “mass of pups.” Calculations to the side of the pups were not performed; however, these are not expected to be significantly different to the mean frontal exposure SAR values as the cross‐sectional area of the “mass of pups” perpendicular to the incident field is similar. For vertically polarized electric field exposure, field values varying between 147 and 55.9 V/m produced a mean whole‐body SAR of 0.5 W/kg in a single pup between 0.9 and 29 g, respectively, within the “mass of pups” model. Electric field values varying between 208 and 79.1 V/m produced a mean whole‐body SAR of 1 W/kg in a single pup between 0.9 and 29 g respectively, within the same “mass of pups” model.

Animals were exposed to LTE signals for 30 min/day, 5 days/week from gestation day 13.5 to gestational day 18.5, and from postnatal day 3 to postnatal day 21 (weaning) at a SAR of 0.5 or 1 W/kg.

Each animal was weighed every day before exposure (the 30‐min exposures were performed between 9 and 10 am), and the electric field intensity was adjusted to ensure that the mean exposure of that group of animals was at the nominal value. It was necessary to expose pups without their mothers to ensure that the nominal SAR could be achieved, and it was decided that pups should not be removed from their mothers until 3 days old. Other pregnant mothers and pups were sham‐exposed by undergoing the exact same procedures, except that the electric field strength in the GTEM was reduced to 0. The 30‐min daily exposure of mice was unlikely to cause additional stress from reduced mobility inside the small opaque boxes, or separation stress in the pups [Gammie and Stevenson, 2006].

Specially designed polystyrene cages with removable internal partitions were made for the exposure of the pregnant animals and their offspring in the GTEM. These reduced the amount of movement the animals could make, especially in the vertical direction, but they did not completely restrain the animals; small pups were free to move, but they tended to nest in a group. The internal partitions were sequentially removed to allow for growth of the animals. The previous mapping of the GTEM cell indicated that there was some variation in the distribution of the electric field strength inside the chamber. The field variation at both 1,840 and 1,850 MHz was known in 10 cm steps and was averaged for the area occupied by the cage. To minimize the variation of the electric field strength inside each cage, as well as between cages, the cages were placed inside the GTEM cell at a specific height where field variation had been found to be minimal, but the electric field strength was high enough to get the required SAR levels. Four measurements were made, one at each of the locations of the mice, plus a measurement at the center. These were then averaged. The variation for a target of 10 V/m was 11.31–8.7 V/m. Moreover, regarding the position of cages in the horizontal plane, an array of cells in the central zone of the exposure area was chosen from the 10‐cm step matrix of the field map. Figure 6 shows (a) block diagram for LTE signal generation, and (b) photograph showing the position of the polystyrene mouse cages during exposure with the probe used for monitoring the electric fields. The foam blocks under the cages were made of a low dielectric constant branded material (EccoStock SH 2; Emerson & Cuming, Randolph, MA), and (c) screengrab of a digitally demodulated LTE downlink signal was used in this study. It was generated using a Keysight N9080A LTE‐FDD measurement application on an Agilent N9020A MXA Signal Analyzer. The mice were exposed without access to food and water, in order to minimize the perturbation of the RF field.

### Home Cage

When the male mice were 10 weeks of age, they were individually and randomly placed into PhenoTyper home cages (Noldus Information Technologies, Wageningen, The Netherlands). Each home cage consists of a 45 × 45 × 56 cm white base floor and a top unit with an integrated infrared‐sensitive camera and a source of a white spotlight. Inside the cage, a white shelter was placed in one of the corners with two distinct entrance holes. The food and water were available ad libitum. Two weeks were allowed for habituation to the cages and to determine the effects of early‐life exposure on later life. Data collection began from the age of 12 weeks, when the mice were sexually mature young adults, and continued to 28 weeks of age, when the mice were mature adults.

The cages were self‐contained with drinking water, food supply, shelter, and an activity wheel (PhenoWheel; Noldus Information Technologies).

To reduce disturbance to the animals, white highly absorbent bedding (ALPHA‐dri; Shepherd Specialty Papers, Kalamazoo, MI) was replaced every 4–6 weeks, and some animals were weighed at the same time to provide measurements of body mass (*n* = 3).

### Behavioral Analysis and Statistics

Tracking and behavioral data analysis was performed using Ethovision v 9.0 software (Noldus Information Technologies). Animals were monitored on a 24‐h basis to collect automatic data for the number of licks against the spout of the water bottle and the number of running wheel cycles. A camera in the top of the cage tracked spontaneous activities, using center‐point tracking. These were overall distance moved, cumulative moving duration, cumulative duration in the shelter, frequency of visits to the shelter, frequency of non‐active episodes, and number of visits to the feeder zone at a sample rate of 12 samples per second. Data were continuously acquired for 13 weeks (except for 6 h a week for housekeeping duties).

The base of the floor of the cage was virtually divided by Ethovision software into zones for feeding and for the shelter. Figure 7 shows the location of the feeder zone and the shelter zone used for analysis purposes. Other zones are for illustrative purposes only. Data for number of licks made against the water spout and revolutions of the running wheel were automatically collected by the Lickometer and the PhenoWheel (both Noldus Information Technologies).

All statistical analyses were carried out using Minitab 18 (Minitab, State College, PA). Datasets were tested for normality using the Anderson‐Darling normality test, using *P* < 0.05, and were from a normally distributed population, so normal assumptions were appropriate for this data. The general linear model (GLM) for analysis of variance (ANOVA) was used, which uses a least‐squares regression approach, to determine differences between treatment groups, using factors of treatment group and age. Two‐sided 95% confidence intervals and the adjusted sum of squares were used in the GLM. *α* was always set at 0.05. All results are expressed as mean ± standard error of the mean. Post hoc tests were undertaken for endpoints that had a *P* ≤ 0.05 using pair‐wise Fishers *t* tests, which control the individual confidence level.

## RESULTS

Exposure to RF fields at either SAR had no discernible adverse effects on pregnancy or litter size compared with sham‐exposed controls. There were no gross external malformations or abnormal morphological changes in any of the offspring. Daily health and welfare inspection of the animals did not reveal any adverse effects.

### Body Mass

**Figure 2:**
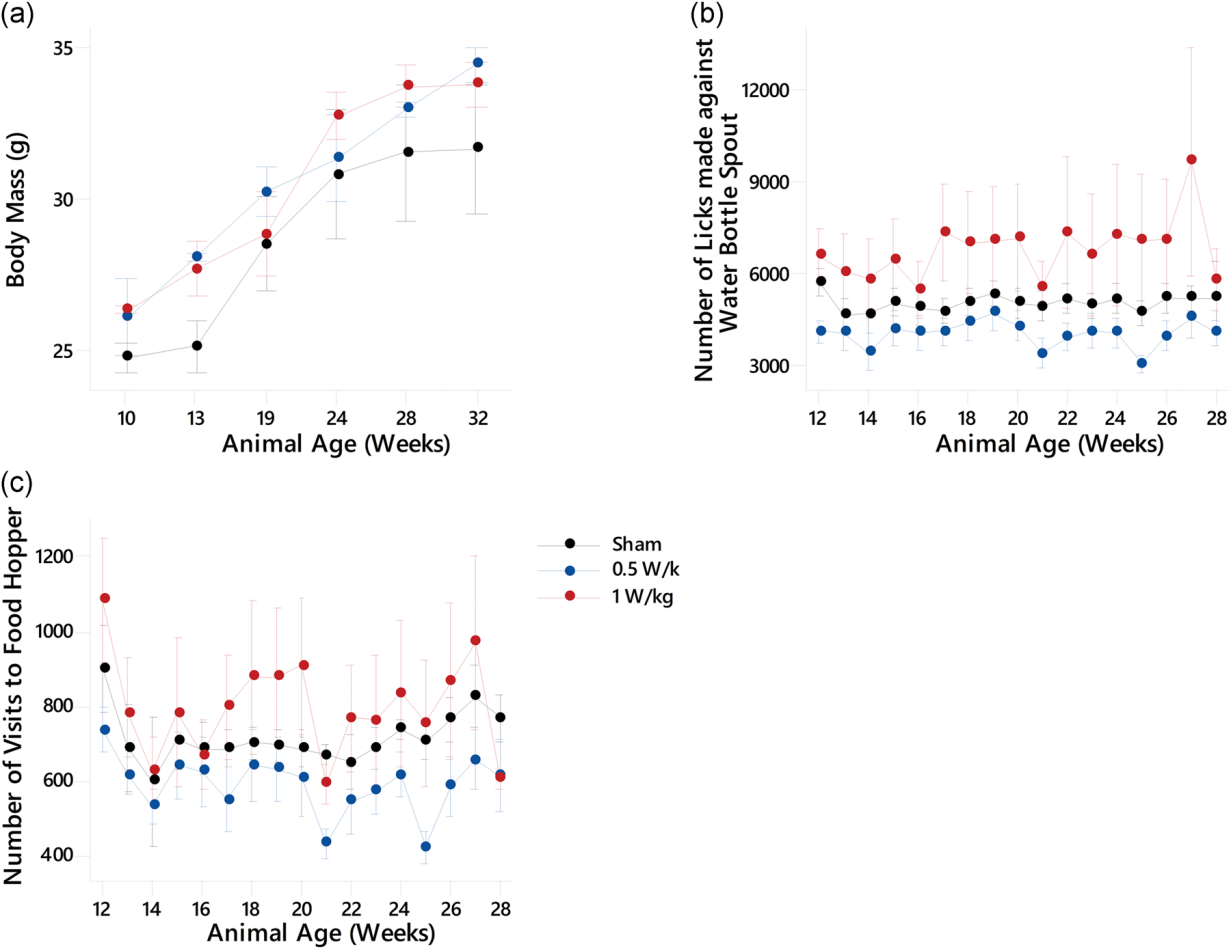
Appetitive behavior after prenatal and early‐life exposure to radiofrequency (RF) fields. (**a**) Animal body mass (*n* = 3 per experimental group), (**b**) number of licks made against the spout of the water bottle, and (**c**) number of visits to the food hopper. See Table 2 for significant differences between groups. For all experimental groups *n* = 5 or 6 except for weeks 14, 21, and 28 where *n* = 3. All data are presented as mean ± standard error of the mean.

Mice were weighed using a top pan balance (XT3200D; Precisa UK, Newport Pagnell, UK) every 4–6 weeks between 10 and 32 weeks of age. As expected, all mice gained body mass during the study (Fig. 2a). Exposure had a significant effect on body mass as found by GLM ANOVA, (*F*(2,35) = 4.25, *P* ≤ 0.022). Animals exposed at 0.5 and 1 W/kg both showed an increased body mass when compared with sham‐exposed animals (95% CI [0.484, 3.108], *P* ≤ 0.008 and 95% CI [0.484, 3,065], *P* ≤ 0.008), respectively.

**Table 2:** Summary of Changes in Appetitive and Activity Behaviors Following Pre‐Natal and Early Life Exposure to RF Fields Compared With Sham Exposed Mice

Endpoint	0.5 W/kg	1 W/kg
Licks against water spout	↓	↑
Number of visits to feeder	↓	↓
Number of PhenoWheel revolutions	ns	↑
Cumulative moving duration	↓	↑
Overall distance moved	↓	ns
Number of non‐active episodes	↓	↑
Number of visits to shelter	↑	↑
Cumulative time in shelter	↑	↑

Arrows indicate a significant increase or decrease (*P* < 0.05) in the endpoints, ns indicates not significantly different. See text for full description of endpoints.

### Drinking

Drinking was assessed by automatically counting the number of licks made against the spout of the water bottle (Fig. 2b). GLM ANOVA showed that there was a significant effect of exposure (*F*(2,212) = 19.04, *P* ≤ 0.000). Compared with sham‐exposed mice, animals exposed at 0.5 W/kg showed a significantly decreased number of licks (95% CI [−1,932, −98], *P* ≤ 0.03), and animals exposed at 1 W/kg showed a significantly increased number of licks (95% CI [835, 2,643], *P* ≤ 0.000). Furthermore, animals exposed at 0.5 W/kg also made a significantly decreased number of licks compared with the mice exposed at 1 W/kg (95% CI [1,862, 3,645], *P* ≤ 0.000).

### Feeding Behavior

Feeding behavior was assessed by the number of visits to the feeder zone of the home cage (Fig. 2c). Generally, exposure at 0.5 or 1 W/kg decreased feeding behavior throughout the study period. GLM ANOVA showed that there was a significant effect of exposure for the number of visits to the feeder zone over time (*F*(2,212) = 10.89, *P* ≤ 0.000). Mice exposed at 0.5 W/kg had a significantly decreased number of visits when compared with the sham‐exposed mice (95% CI [−216.3, −34.9], *P* ≤ 0.007), or the animals exposed at 1 W/kg (95% CI [119.5, 295.8], *P* ≤ 0.000). In addition, the 1 W/kg exposed group had an almost significantly increased number of visits to the feeder zone when compared with the sham‐exposed group (95% CI [−7.4, 171.5], *P* ≤ 0.072).

### Locomotor Behavior

Activity and locomotion were assessed by measuring the total number of revolutions of the running wheel (PhenoWheel) performed by the mice in the home cage, the overall distance moved, cumulative moving duration, and number of active episodes.

### PhenoWheel

**Figure 3:**
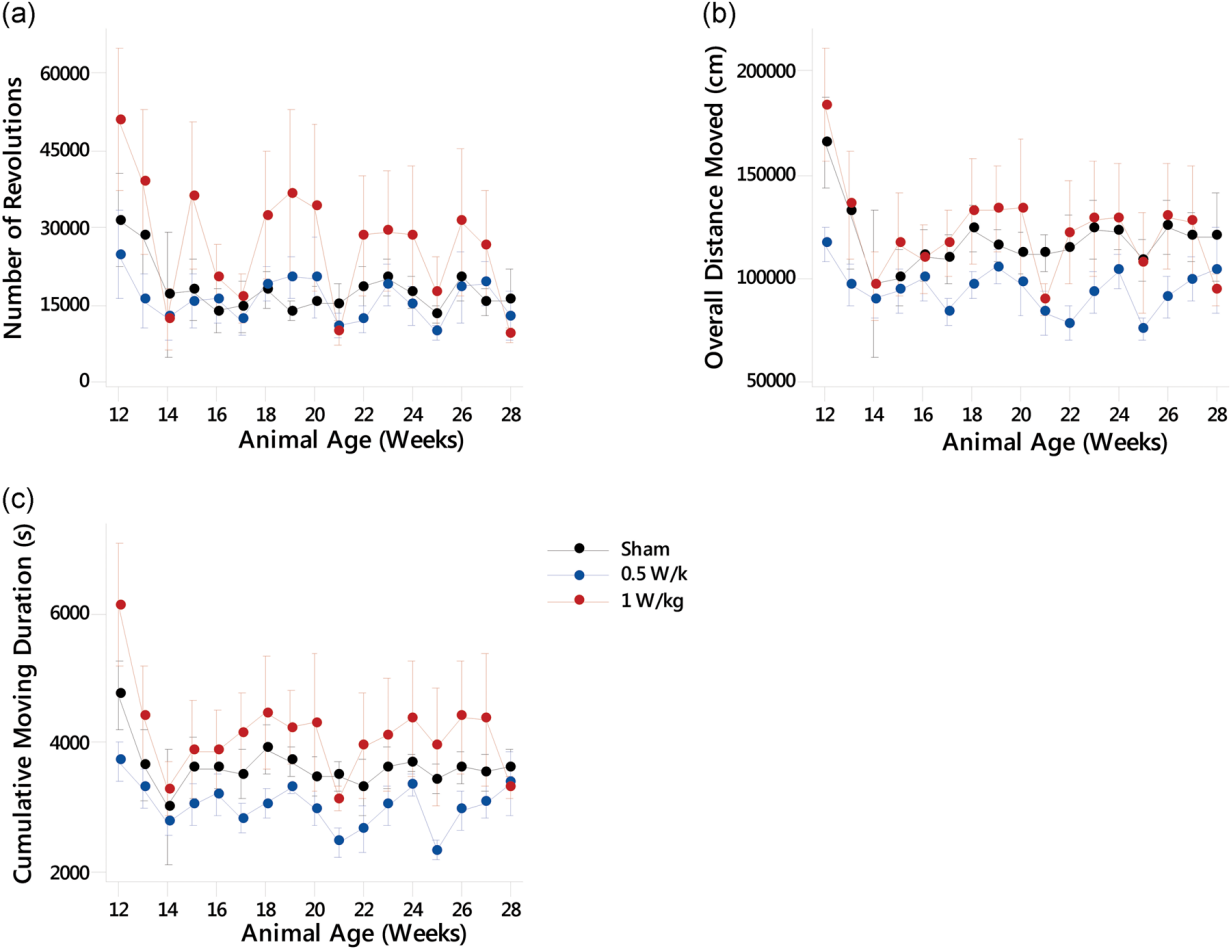
Locomotor behavior after prenatal and early‐life exposure to radiofrequency (RF) fields. (**a**) Number of revolutions of the PhenoWheel, (**b**) overall distance moved (cm), and (**c**) cumulative moving duration (seconds). See Table 2 for significant differences between groups. For all experimental groups *n *= 5 or 6 except for weeks 14, 21, and 28 where *n* = 3 due to data loss. All data are presented as mean ± standard error of the mean.

The number of revolutions on the PhenoWheel (Fig. 3a) was found to vary significantly between treatment groups as found by a GLM ANOVA (*F*(2,199) = 6.42, *P* ≤ 0.002). The 1 W/kg exposed group performed a significantly higher number of revolutions than either the sham‐exposed group (95% CI [2,614, 15,224], *P* ≤ 0.006) or the 0.5 W/kg exposed group (95% CI [4,281, 17,113], *P* ≤ 0.001).

### Overall Distance Moved

When the overall distance moved by the mice in the home cages was studied (Fig. 3b), GLM ANOVA showed that there was a significant effect of exposure (*F*(2,212) = 10.09, *P* ≤ 0.000). The distance moved by mice exposed to 0.5 W/kg was significantly less than either the sham‐exposed mice (95% CI [−37,309, −10,246], *P* ≤ 0.001) or the 1 W kg^−1^ exposed mice (95% CI [14,937, 41,246], *P* ≤ 0.000).

### Cumulative Moving Duration

The total duration of movement in the home cage was also measured (Fig. 3c), and GLM ANOVA showed a significant effect of exposure (*F*(2,212) = 14.45, *P* ≤ 0.000). It was found that the cumulative moving duration was significantly reduced in mice that were exposed to 0.5 W/kg compared with sham‐exposed animals (95% CI [−1,015, −178], *P* ≤ 0.005) or mice exposed at 1 W/kg (95% CI [706, 1,520], *P* ≤ 0.000). Mice exposed to 1 W/kg also showed an increased cumulative moving duration compared with the sham‐exposed group (95% CI [104, 929], *P* ≤ 0.014).

### Rest Behavior

In order to assess rest behavior, the number of non‐active episodes exhibited by the mice, number of visits to the shelter in the home cage, and cumulative time in the shelter were collected for 17 weeks and averaged over consecutive 6.5‐day periods for each treatment group.

### Number of Non‐Active Episodes

**Figure 4:**
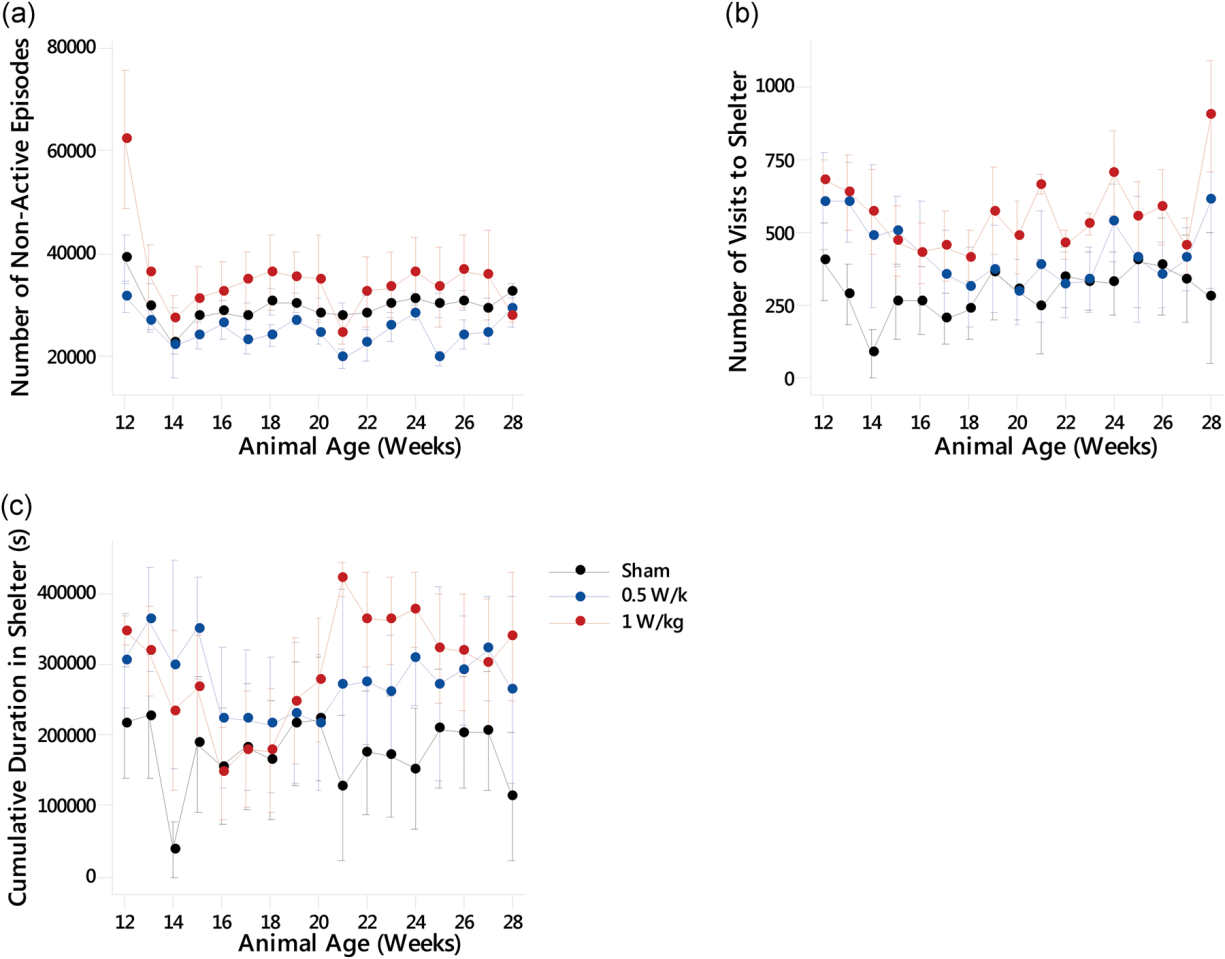
Rest behavior after prenatal exposure to radiofrequency (RF) fields. (**a**) Number of non‐active episodes, (**b**) number of visits to the shelter, and (**c**) cumulative duration in shelter. See Table 2 for significant differences between groups. For all experimental groups *n* = 5 or 6 except for weeks 14, 21, and 28 where *n* = 3 due to data loss. All data are presented as mean ± standard error of the mean.

The number of episodes when mice were quiescent and did not actively explore or forage about the cage (defined as an animal having a velocity of 1.75 cm/s or less) were measured (Fig. 4a). GLM ANOVA showed that there was a significant effect of exposure found (*F*(2,212) = 14.93, *P* ≤ 0.000). It was found that the number of non‐active episodes in the 0.5 W/kg exposed group was significantly decreased compared with the sham‐exposed animals (95% CI [−8,487, −1,116], *P* ≤ 0.011) and the animals exposed at 1 W/kg (95% CI [6,342, 13,508], *P* ≤ 0.000). The animals exposed at 1 W/kg had a significantly increased number of non‐active episodes compared with the sham‐exposed animals (95% CI [1,489, 8,758], *P* ≤ 0.006).

**Figure 5:**
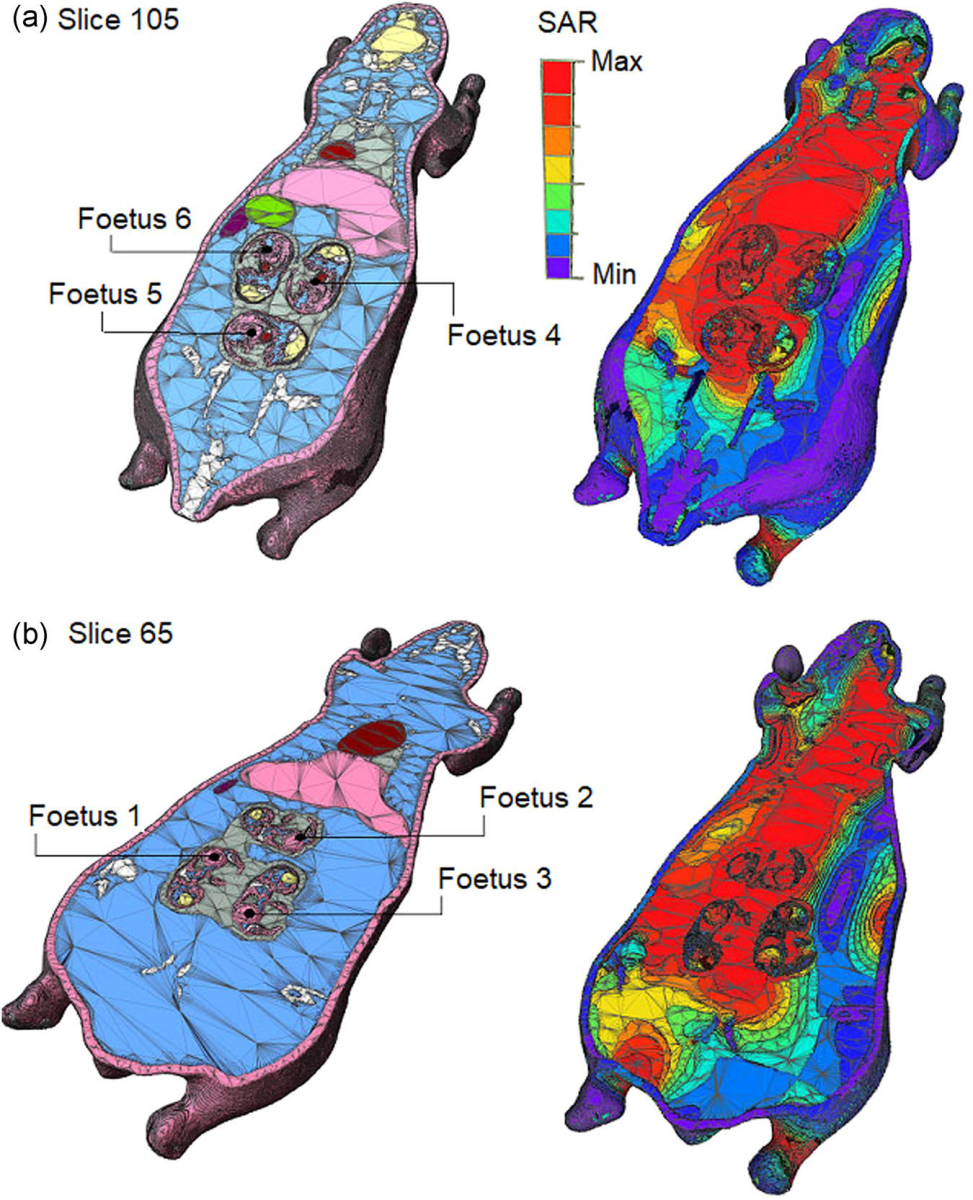
Anatomical and specific energy absorption rate (SAR) distribution images for axial slices of the pregnant mouse model. The incident field is to the left side of the mouse and horizontally polarized. Slices 65 (**a**) and 105 (**b**) are shown, providing cross‐sections of the six fetuses.

**Figure 6:**
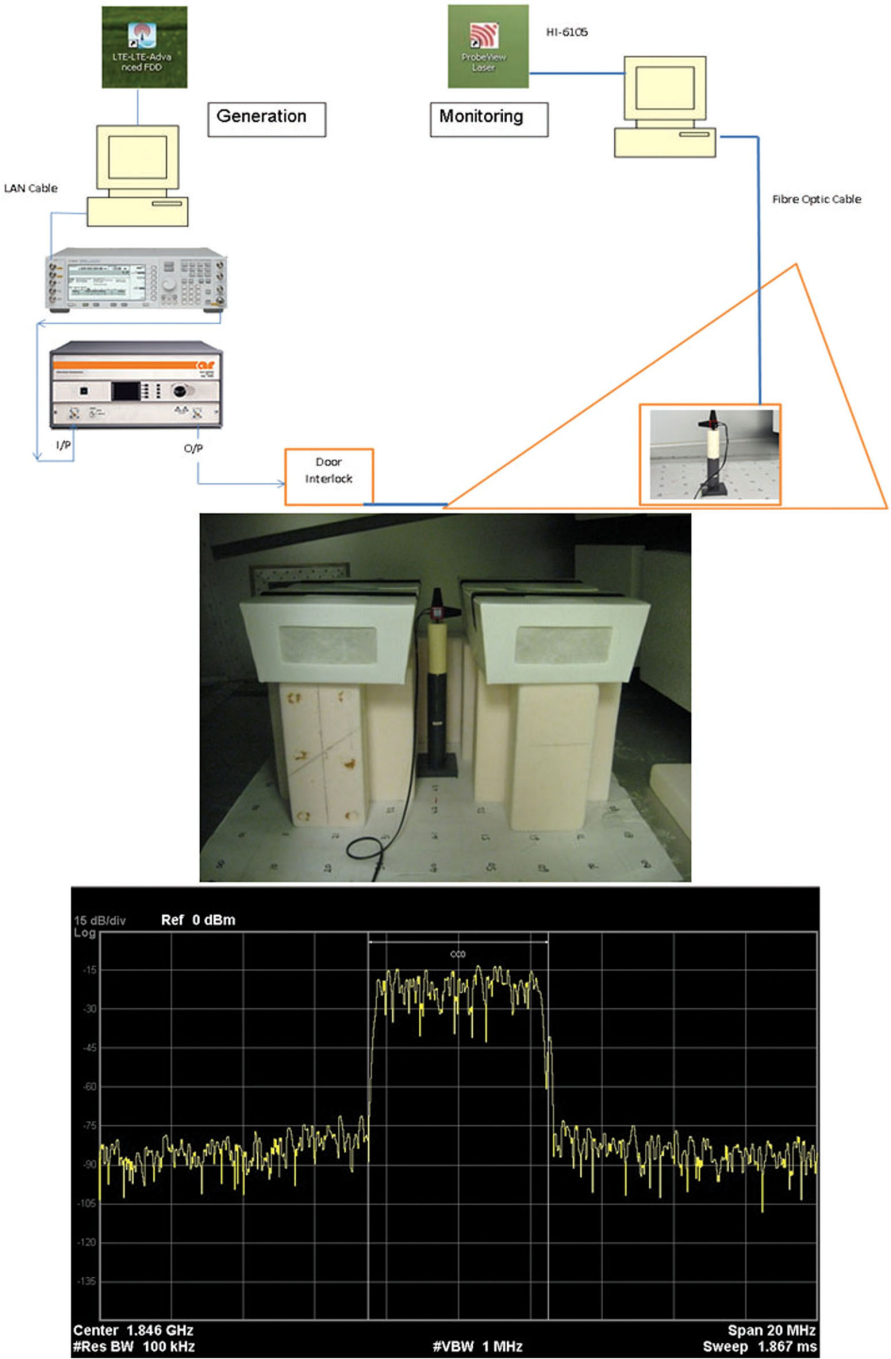
Experimental setup. (**a**) Block diagram for long term evolution (LTE) signal generation and monitoring. (**b**) Photograph showing position of the polystyrene mouse cages during exposure. Foam blocks under the cages are made of a low dielectric constant material. (**c**) Screengrab of a digitally demodulated LTE downlink signal as used in this study. It was obtained using a Keysight N9080A LTE‐FDD measurement application, on an Agilent N9020A MXA Signal Analyzer.

**Figure 7:**
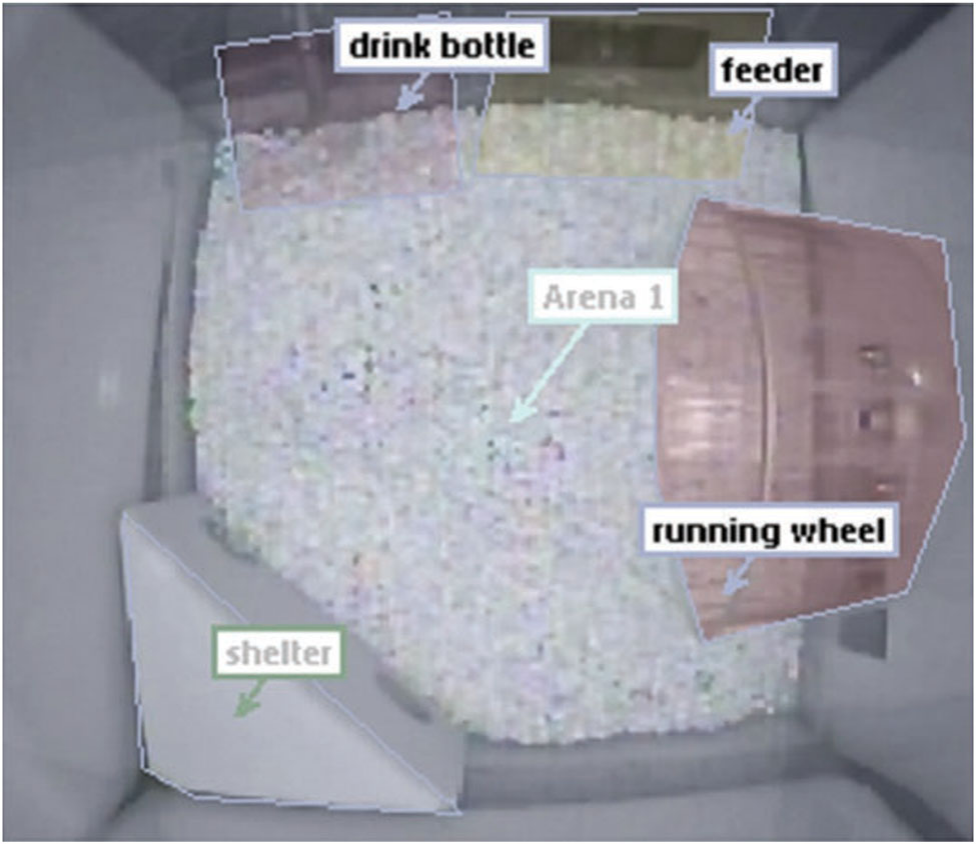
Photograph of the inside of a home cage showing features and analysis zones. A mouse was housed in a home cage for 13 weeks to collect behavioral data. The feeder zone and shelter zone were used for analysis but the other zones are for illustrative purposes only.

### Number of Visits to the Shelter

When the number of visits to the shelter was studied (Fig. 4b), GLM ANOVA showed that there was a significant effect of exposure (*F*(2,212) = 15.83, *P* ≤ 0.000). The 0.5 W/kg exposed group had an increased number of visits compared with the sham‐exposed group (95% CI [39.6, 227], *P* ≤ 0.006) and 1 W/kg exposed group (95% CI [39.3, 221.5], *P* ≤ 0.005). The 1 W/kg exposed group also had a significantly increased number of visits compared with the sham‐exposed group (95% CI [1,713, 356.1], *P* ≤ 0.000).

### Cumulative Duration in the Shelter

The GLM ANOVA showed that the cumulative duration in the shelter (Fig. 4c) was affected by exposure (*F*(2,212) = 8.902, *P* ≤ 0.000). Exposure to 0.5 or 1 W/kg resulted in a longer cumulative duration in the shelter when compared with the sham‐exposed animals (95% CI [40,946, 161,710], *P* **< **0.001 and 95% CI [60,000, 179,090], *P* ≤ 0.000, respectively).

## DISCUSSION

In this study, prenatal and early postnatal exposure to RF fields caused long‐lasting changes in some aspects of mouse behavior. We observed that appetitive behavior, as well as locomotor and rest behaviors, were altered by early‐life exposure to RF fields. The changes that we observed in our study varied with exposure (expressed as whole‐body averaged SAR) and were persistent over the 6‐month time period of the experiment, but there was no consistent exposure‐response relationship. We believe that this is the first time that such changes in adult behavior has been shown after an early‐life exposure. However, while the present results provide useful input into any risk assessment regarding the impact of RF fields on human health, the SARs we used were above the restrictions recommended by ICNIRP for whole‐body exposures [ICNIRP, 1998].

Significant increases in body mass were observed in the offspring of both groups of animals exposed to the RF fields. Although these differences were consistent throughout the study, this result must be considered preliminary since the numbers of animals in these treatment groups were small. Previously, many but not all studies suggest that the exposure of pregnant rodents of 1–5 GHz at up to 4 W/kg had no significant effect on body mass of offspring [Sambucci et al., 2011; Poulletier de Gannes et al., 2012; Othman et al., 2017; Shirai et al., 2017]. Therefore, the observed increase in body mass reported here requires further investigation. In contrast, Wyde et al. [2018] reported that pre‐natal and early exposure of rats to GSM or CDMA signals at up to 6 W/kg for 9 h/day reduced litter mass (up to 9%). These differences persisted into early lactation with continuing daily exposure to either signal at the highest SAR, but these gradually lessened as lactation progressed, and no further field‐dependent differences in body mass were observed for the remainder of the two‐year study. In addition, some older studies have suggested that thermogenic exposures may be associated with a reduction in body mass. For example, Berman et al. [1984] reported a very transient decrease in rat body mass after exposure to 2.45 GHz at 16.5 W/kg and Jensh [1997] also reported a temporary body mass reduction in rats exposed to 6 GHz at about 7 W/kg. The usefulness of these studies in rats using high SARs is less relevant to the present results. Of more relevance and more recently, it has been reported that pre‐natal exposure of female rats to pulsed 2.45 GHz at 0.1 W/kg resulted in a reduction in daily body mass gain [Sangun et al., 2015]. Therefore, the observed increase in body mass reported here requires further investigation, and the lack of any dose‐response is intriguing.

The two field‐exposed groups could be distinguished on the basis of their observable behavior in the home cage, and generally, exposure at 0.5 W/kg had an inhibitory effect, whereas exposure at 1 W/kg had a stimulatory effect on behavior. For example, exposure at the lower SAR resulted in decreased drinking behavior and decreased locomotor activities (as measured by cumulative moving duration and overall distance moved). In contrast, exposure at the higher SAR resulted in increased drinking behavior and increased locomotor activity (as measured by the number of revolutions of the PhenoWheel). However, some changes in behavior were common to both field‐exposed groups: compared with sham‐exposed animals, these groups showed an increased number of visits to the shelter and an increased cumulative duration in the shelter, as well as increased periods of non‐activity (measured on the home cage floor). This suggests that although the mice exposed at 1 W/kg were more active in the PhenoWheel, they also spent more time at rest (and possibly sleep) than sham‐exposed control animals.

Other studies have demonstrated that field‐induced increased activity in mice after exposure using a mobile phone handset [Aldad et al., 2012] at an SAR estimated to 1.6 W/kg (although actual exposure was probably substantially lower) [Hansson Mild et al., 2012]. In addition, long‐term exposure (12 weeks) of adult mice to an RF field at a higher SAR (4 W/kg) has also been reported to induce increased activity, in terms of distance moved and the duration of movement [Kim et al., 2017]. The mice in our study were exposed using a well‐defined and controlled exposure system over a period of 24 days in total and at a much lower SAR, suggesting that younger mice with developing nervous systems may be more susceptible to the effects of RF fields than mature animals. The possibility that younger animals could be more susceptible to the effects of RF fields has been expressed for many years [IEGMP, 2000], though the counter‐argument that exposure to RF electromagnetic fields do not affect brain development or health in children has also been explored. It was suggested that such effects cannot be ruled out because there are limited studies and those that are available tend to focus on older children [HCN, 2011].

It is pertinent to study the observed changes in appetitive behaviors in more detail to examine the underlying mechanism for these changes. It is known that swallowing and ingestive behaviors develop in utero [Ross and Nijland, 1998], and it is possible that the development of these behaviors was affected by exposure to RF fields. Feeding behavior can be linked to metabolic energy requirements but may also be influenced by hippocampal‐dependent mnemonic function [Kanoski and Grill, 2017]; however, no correlation with ingestion and activity were observed in our results. It is feasible that the episodic memories of drinking and feeding by the mice were somehow disrupted by alterations to the developing hippocampal neurons that are responsible for the control of these behaviors. Deficits in memory as a result of altered hippocampal function has been described in rodents [Fortin et al., 2004], and hippocampal function has been considered to be sensitive to RF field exposure [Sienkiewicz and van Rongen, 2019].

Further study is necessary to examine this possibility and to try and determine the underlying reason for the observed changes. Since behavior was measured a few weeks after all exposures had been completed, any simple perceptual or thermal‐based explanation of these effects seems untenable, as does an explanation based on the presence of some unidentified stress in the animals’ environment, because it is unclear how any single factor could cause behavioral changes in opposite directions in different groups. In order to avoid systematic errors, all animals were treated in an identical manner except for actual treatment, animals were randomly allocated to the home cages to prevent some bias based on position within the laboratory, and all data were collected and analyzed without knowledge of the exposure status of the animals. It was also necessary to collect the data in two separate equal‐sized experimental runs (with three animals per treatment group in each), and both runs yielded the same result for each type of treatment. In contrast, the differences in behavior were not observed in pilot studies with unexposed animals. Finally, the consistency and persistency of the observed changes over the course of the experiment suggests that random chance also seems unlikely, and suggests the experimental protocol itself is not responsible for the observed changes. Overall, this suggests that exposure to the RF fields was responsible for the observed changes. In this regard, it would be valuable to examine the brains of exposed animals to seek evidence of concordant changes in neuronal structure, such as alterations in synapse structure, neurotransmitter status, cell recruitment, or breakdown in the blood‐brain barrier. However, since the mice received a whole‐body exposure, there could also be effects in other areas of the central nervous system or in systems outside the brain which may have contributed to the behavioral outcomes. Such abscopal mechanisms have been attributed to prenatal exposures to ionizing radiation in humans [Mole, 1990].

In summary, prenatal and early‐life exposure of male mice to pulsed 1,846 MHz RF fields simulating LTE downlink signals at a whole‐body averaged SAR of 0.5 or 1 W/kg resulted in consistent and long‐lasting changes in drinking and eating behavior, as well as locomotor and rest behaviors. Further work is required to determine the mechanism(s) responsible for these observations.
